# Crystal structure of 2-(thio­phen-3-yl)ethyl pyrene-1-carboxyl­ate

**DOI:** 10.1107/S2056989015020873

**Published:** 2015-11-11

**Authors:** Bianca X. Valderrama-García, Reyna Reyes-Martínez, Simón Hernández-Ortega, David Morales-Morales, Ernesto Rivera

**Affiliations:** a Instituto de Investigaciones en Materiales, Universidad Nacional Autónoma de México, Coyoacán 04510, México D.F., Mexico; b Instituto de Química, Universidad Nacional Autónoma de México, Coyoacán 04510, México D.F., Mexico

**Keywords:** crystal structure, pyrene, thio­phene, excimers, exciplexes, hydrogen bonding, S⋯π contacts

## Abstract

In the title compound, C_23_H_16_O_2_S, the thio­phene group is rotationally disordered into two fractions almost parallel to each other, with occupation factors of 0.523 (7) and 0.477 (7), and subtending dihedral angles of 10.5 (5) and 9.3 (5)°, respectively, to the thio­phene group. The mol­ecules are held together by weak C—H⋯O and C—H⋯π hydrogen bonds, producing a laminar arrangement, which are further connected in a perpendicular fashion by S⋯π contacts [S⋯centroid = 3.539 (8) and 3.497 (8) Å]. In spite of the presence of the entended pyrene group, the structure does not present any parallel π–π stacking inter­actions. The structure was refined as an inversion twin.

## Related literature   

For optical and electronic properties of pyrene compounds, see: Hrdlovič & Lukáč (2000 ▸); Winnik (1993 ▸); Kim *et al.* (2008 ▸). For use of pyrenes as sensors, see: Basu & Rajam (2004 ▸); Chmela *et al.* (2005 ▸). For applications of thio­phenes, see: Perepichka *et al.* (2005 ▸); Abd-El-Aziz *et al.* (2013 ▸). For a previous report of meth­oxy­pyrene, see: Morales-Espinoza *et al.* (2015 ▸). For S⋯π inter­actions, see: Mooibroek *et al.* (2008 ▸).
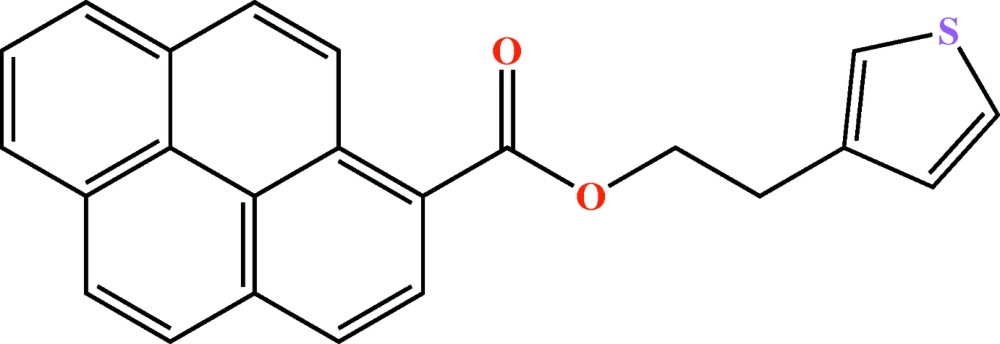


## Experimental   

### Crystal data  

C_23_H_16_O_2_S
*M* *_r_* = 356.42
Orthorhombic, 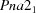

*a* = 12.020 (9) Å
*b* = 7.576 (6) Å
*c* = 18.521 (14) Å
*V* = 1687 (2) Å^3^
*Z* = 4
Mo *K*α radiation
μ = 0.21 mm^−1^
*T* = 298 K
0.30 × 0.23 × 0.17 mm

### Data collection  

Bruker APEXII CCD diffractometer
Absorption correction: multi-scan (*SADABS*; Bruker, 2012 ▸) *T*_min_ = ?, *T*_max_ = ?
11964 measured reflections
3116 independent reflections
2546 reflections with *I* > 2σ(*I*)
*R*_int_ = 0.162

### Refinement  

*R*[*F* ^2^ > 2σ(*F* ^2^)] = 0.057
*wR*(*F* ^2^) = 0.148
*S* = 1.05
3116 reflections
256 parameters
56 restraints
H-atom parameters constrained
Δρ_max_ = 0.19 e Å^−3^
Δρ_min_ = −0.16 e Å^−3^
Absolute structure: Refined as an inversion twin.
Absolute structure parameter: 0.3 (2)

### 

Data collection: *APEX2* (Bruker, 2012 ▸); cell refinement: *SAINT* (Bruker, 2012 ▸); data reduction: *SAINT* (Bruker, 2012 ▸); program(s) used to solve structure: *SHELXS97* (Sheldrick 2008 ▸); program(s) used to refine structure: *SHELXL2014* (Sheldrick, 2015 ▸); molecular graphics: *SHELXTL* (Sheldrick 2008 ▸); software used to prepare material for publication: *PLATON* (Spek, 2009 ▸) and *DIAMOND* (Brandenburg, 2006 ▸).

## Supplementary Material

Crystal structure: contains datablock(s) I, global. DOI: 10.1107/S2056989015020873/bg2569sup1.cif

Structure factors: contains datablock(s) I. DOI: 10.1107/S2056989015020873/bg2569Isup2.hkl

Supporting information file. DOI: 10.1107/S2056989015020873/bg2569Isup3.cml

**Fig. 1.**
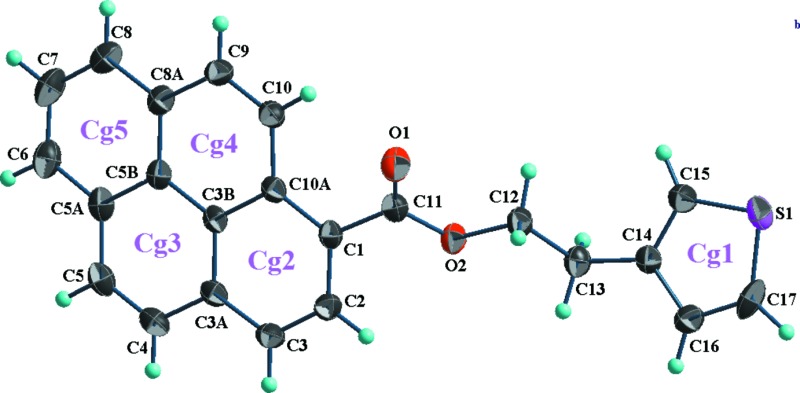
The molecular structure of the title compound, showing the atom labelling. Displacement ellipsoids are drawn at the 30% probability level. Only the major fraction of the disordered thiophene has been drawn.

. DOI: 10.1107/S2056989015020873/bg2569fig1.tif
The mol­ecular structure of the title compound, showing the atom labelling. Displacement ellipsoids are drawn at the 30% probability level. Only the major fraction of the disordered thio­phene has been drawn.

**Fig. 2.**
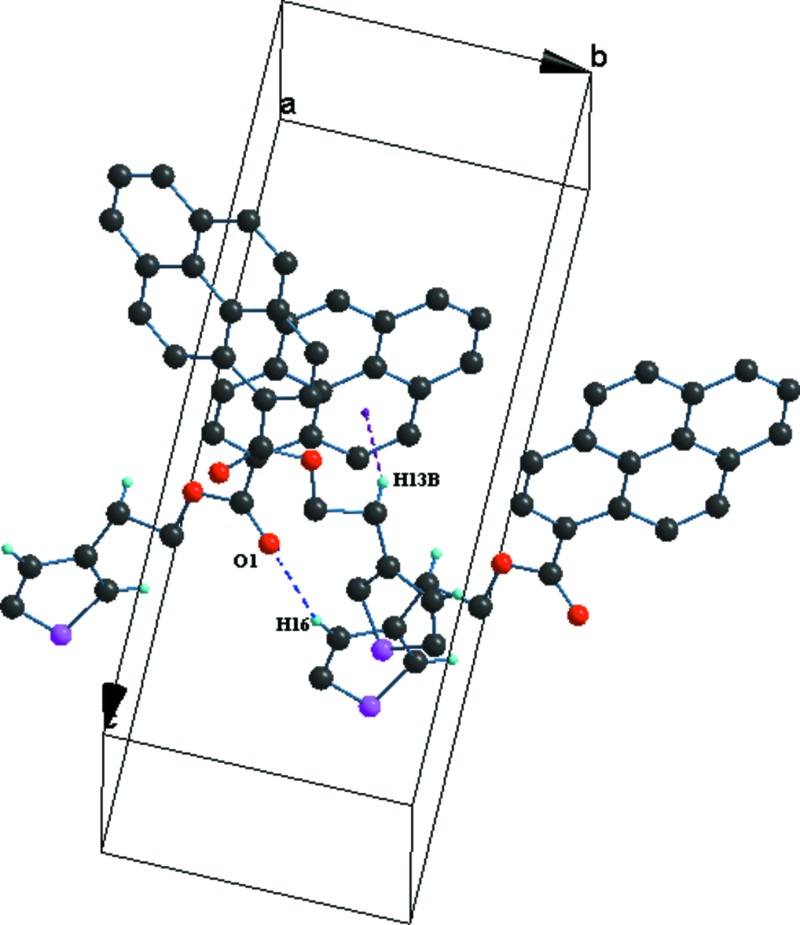
A partial view of crystal packing of the title compound showing C—H···O and C—H···Cg interactions, drawn as dashed lines. Only H atoms involved in hydrogen bonding have been included for clarity.

Cg . DOI: 10.1107/S2056989015020873/bg2569fig2.tif
A partial view of crystal packing of the title compound showing C—H⋯O and C—H⋯*Cg* inter­actions, drawn as dashed lines. Only H atoms involved in hydrogen bonding have been included for clarity.

**Fig. 3.**
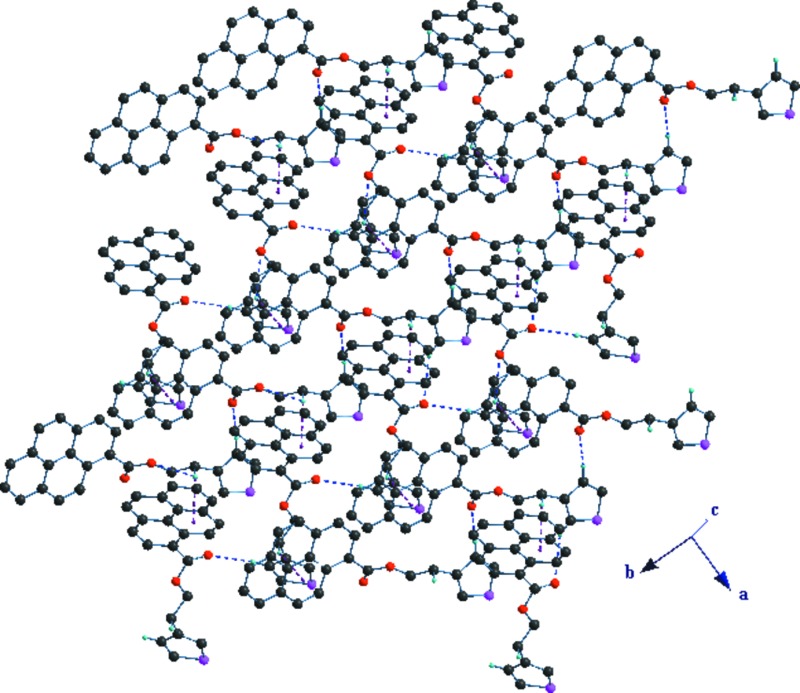
A view of the crystal packing of the title compound, with the hydrogen bonds shown as dashed lines. Only H atoms involved in hydrogen bonding have been included.

. DOI: 10.1107/S2056989015020873/bg2569fig3.tif
A view of the crystal packing of the title compound, with the hydrogen bonds shown as dashed lines. Only H atoms involved in hydrogen bonding have been included.

**Fig. 4.**
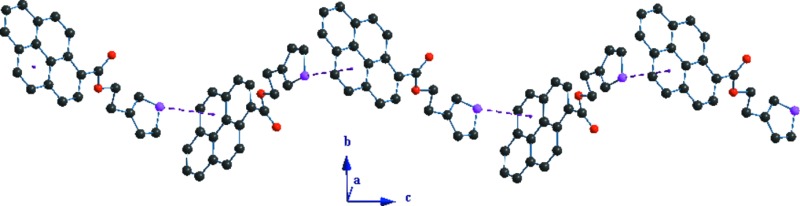
Representation of the S···π interaction (Only major fraction of the disordered thiophene group). Hydrogen atosm omitted.

. DOI: 10.1107/S2056989015020873/bg2569fig4.tif
Representation of the S⋯π inter­action (Only major fraction of the disordered thio­phene group). Hydrogen atosm omitted.

CCDC reference: 1435021

Additional supporting information:  crystallographic information; 3D view; checkCIF report

## Figures and Tables

**Table 1:** Hydrogen-bond geometry (Å, °)

*D*—H⋯*A*	*D*—H	H⋯*A*	*D*⋯*A*	*D*—H⋯*A*
C16—H16⋯O1^i^	0.93	2.55	3.448 (6)	161
C13—H13*B*⋯*Cg*3^ii^	0.97	2.86	3.776 (5)	155

Symmetry codes: (i) 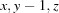; (ii) 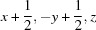.

## supplementary crystallographic information

### S1. Chemical context

Pyrene and their derivatives are well-known for their optical and electronic properties (Hrdlovič & Lukáč, 2000; Winnik 1993). They exhibit long fluorescence lifetimes in non-polar media (ca. 400 ns) in addition to their ability to form homo and hetero-dimmers in excited states (excimers, exciplexes) (Kim *et al.*, 2008). The photophysical properties of pyrene derivatives can be used as sensor for oxygen (Basu & Rajam, 2004) and as monitor for polymerization reactions (Chmela *et al.*, 2005). The fluorescence studies of pyrenes as sensor are based in processes of electron transference, changes of wavelength of higher emission and the formation of excited states (excimers, exciplexes).

The preparation of thio­phene derivatives with fluorescent properties has been studied in order to obtain polythio­phenes. The polymers of thio­phenes are a class of linear conjugated polymers characterized by their versatility and are used as materials for electronic and optoelectronic applications (Perepichka *et al.*,2005) (Abd-El-Aziz *et al.*, 2013).

In this context, we present the crystal structure of a pyrene functionalized with a thio­phene moiety.

### S2. Structural commentary

The molecular structure of the title compound is shown in Fig. 1. The compound shows rotational disorder at the thio­phene group into two planar, almost parallel moieties (See Refinement section). The thio­phene ring presents a *trans* configuration with respect to the carboxyl­atepyrene group with a torsion angle C14—C13—C12—O2 of -170.9 (3) °. Bond distances are in agreement with those reported for similar organic compounds (Allen *et al.*, 1987).

### S3. Supra­molecular features

Based on the distances obtained using PLATON (Spek, 2009), the crystal packing is the result of weak C—H···O and C—H···π inter­molecular inter­actions, reported in Table 2 and shown in Fig 2, which define laminar arrangements (Fig 3).

Additionally, an S···π inter­action is found which completes the supra­molecular packing. Due to disorder, this inter­action is split into two, viz., S1···Cg3^iii^ and S1A···Cg5^iii^ (iii: 1-x,-y,1/2+z; Cg codes as in Fig 1), with S···Cg distances of 3.539 (7) and 3.487 (7)Å, respectively.], and extends along the [001] direction (Fig. 4). The strength can be considerate as moderate (Mooibroek *et al.*, 2008). It is noteworthy that the structure does not present any parallel π-π stacking inter­actions, in spite of the presence of the entended pyrene group.

### S4. Database survey

We reported previously the crystal structure of 1-meth­oxy­pyrene (Morales-Espinoza *et al.*,2015) where the crystal packing is governed by π-π and C—H···π inter­actions. A search of the Cambridge Structural Database (CSD, CSD version 5.36 updates Nov 2014) with 1-carboxyl­ate skeleton affords eight organic hits, but none with a thio­phene group.

### S5. Synthesis and crystallization

The title compound, 2-(thio­phen-3-yl) ethyl­pyrene-1-carboxyl­ate, was synthesized from the reaction of 3-thio­phene­ethanol (0.105 g, 0.82 mmol), 1-pyrene­carb­oxy­lic acid (0.302 g, 1.23 mmol), N,N'-Di­cyclo­hexyl­carbodi­imide (DCC) (0.507 g, 2.46 mmol) and 4-Di­methyl­amino­pyridine (DMAP) (0.250 mg, 2.05 mmol) in CH_2_Cl_2_ (15 mL) at 0°C for 30 min. The resulting mixture was stirred at room temperature for 12 hours under inert atmosphere. The suspension produced was filtered in order to remove the di­cyclo­hexyl­urea (DCU) formed during the reaction, and the filtrate was evaporated under reduced pressure at 45°C. The crude product was purified by column chromatography in silica gel using first a n-hexane/ CH_2_Cl_2_ (2:5) solvent mixture and then pure CH_2_Cl_2_ as eluent to give the desired product as light yellow crystals. Yield: 87%. MS—CI: m/z = 356.0

^1^H NMR (CDCl_3_, 300 MHz, ppm) (Fig. 4): 7.85-9.2 (m, 9H, Py), 7.37 (dd, 1H, H^5^, *J=*4.9, 3.0 Hz), 7.21 (d, 1H, H^2^, *J=*4.7 Hz), 7.15 (dd, 1H, H^4^, *J=*4.9, 1.3 Hz), 4.76 (t, 2H, *J=*6.8 Hz), 3.26 (t, 2H, *J=*6.8 Hz). ^13^C NMR (CDCl_3_, 75 MHz, ppm): 168.09 (C=O), 155.31 (C^2^, Thioph), 138.38 (C^3^, Thioph), 117.89 (C^4^, Thioph), 96.24 (C^1^, Thioph), 134.41, 131.19, 131.09, 130.46, 129.72, 129.51, 128.47, 127.25, 126.40, 126.38, 126.27, 125.91, 124.98, 124.24, 123.71, 121.94 (C_Py_), 65.23 (OCH_2_), 29.94 (CH_2_).

### S6. Refinement

Crystal data, data collection and structure refinement details are summarized in Table 1. The H atoms were included in calculated positions and treated as riding: C—H = 0.93 Å for aromatic H's and C—H= 0.97 for methyl­ene ones. Uiso(H) = 1.2Ueq(C). The rotational disorder of the thio­phene group was modelled with a couple of split positions (S1—C17 and S1A—C17A), with an occupation ratio of 0.523 (7)/0.477 (7). Similarity restraints in distances and displacemnent factors were used for modelling the disordered fraction. The crystals were poorly diffracting, which led to a high Rint.

### Crystal data

**Table:**

C_23_H_16_O_2_S	*F*(000) = 744
*M**_r_* = 356.42	*D*_x_ = 1.404 Mg m^−^^3^
Orthorhombic, *P**n**a*2_1_	Mo *K*α radiation, λ = 0.71073 Å
*a* = 12.020 (9) Å	θ = 2.2–25.6°
*b* = 7.576 (6) Å	µ = 0.21 mm^−^^1^
*c* = 18.521 (14) Å	*T* = 298 K
*V* = 1687 (2) Å^3^	Prism, yellow
*Z* = 4	0.30 × 0.23 × 0.17 mm

### Data collection

**Table:**

Bruker APEXII CCD diffractometer	2546 reflections with *I* > 2σ(*I*)
φ and ω scans	*R*_int_ = 0.162
Absorption correction: multi-scan (*SADABS*; Bruker, 2012)	θ_max_ = 25.6°, θ_min_ = 2.2°
	*h* = −14→14
11964 measured reflections	*k* = −9→8
3116 independent reflections	*l* = −22→22

### Refinement

**Table:**

Refinement on *F*^2^	H-atom parameters constrained
Least-squares matrix: full	*w* = 1/[σ^2^(*F*_o_^2^) + (0.0753*P*)^2^ + 0.2852*P*] where *P* = (*F*_o_^2^ + 2*F*_c_^2^)/3
*R*[*F*^2^ > 2σ(*F*^2^)] = 0.057	(Δ/σ)_max_ < 0.001
*wR*(*F*^2^) = 0.148	Δρ_max_ = 0.19 e Å^−^^3^
*S* = 1.05	Δρ_min_ = −0.16 e Å^−^^3^
3116 reflections	Extinction correction: *SHELXL2014* (Sheldrick, 2015), Fc^*^=kFc[1+0.001xFc^2^λ^3^/sin(2θ)]^-1/4^
256 parameters	Extinction coefficient: 0.014 (3)
56 restraints	Absolute structure: Refined as an inversion twin.
Hydrogen site location: inferred from neighbouring sites	Absolute structure parameter: 0.3 (2)

### Special details

**Table:**

Geometry. All e.s.d.'s (except the e.s.d. in the dihedral angle between two l.s. planes) are estimated using the full covariance matrix. The cell e.s.d.'s are taken into account individually in the estimation of e.s.d.'s in distances, angles and torsion angles; correlations between e.s.d.'s in cell parameters are only used when they are defined by crystal symmetry. An approximate (isotropic) treatment of cell e.s.d.'s is used for estimating e.s.d.'s involving l.s. planes.
Refinement. Refined as a 2-component inversion twin.

### Fractional atomic coordinates and isotropic or equivalent isotropic displacement parameters (Å^2^)

**Table:**

	*x*	*y*	*z*	*U*_iso_*/*U*_eq_	Occ. (<1)
O1	0.4023 (3)	0.3325 (5)	0.6430 (2)	0.0658 (10)	
O2	0.4120 (3)	0.0607 (4)	0.59746 (17)	0.0510 (8)	
C1	0.2701 (3)	0.2387 (6)	0.5549 (2)	0.0420 (10)	
C2	0.1998 (4)	0.0944 (7)	0.5482 (3)	0.0500 (11)	
H2	0.2155	−0.0085	0.5736	0.060*	
C3	0.1074 (3)	0.1005 (6)	0.5046 (3)	0.0492 (11)	
H3	0.0598	0.0039	0.5025	0.059*	
C3A	0.0849 (3)	0.2482 (6)	0.4641 (2)	0.0440 (10)	
C3B	0.1558 (3)	0.3963 (5)	0.4684 (2)	0.0390 (9)	
C4	−0.0076 (4)	0.2540 (6)	0.4150 (3)	0.0523 (12)	
H4	−0.0551	0.1575	0.4115	0.063*	
C5	−0.0260 (4)	0.3965 (8)	0.3746 (3)	0.0552 (12)	
H5	−0.0868	0.3972	0.3435	0.066*	
C5A	0.0444 (4)	0.5480 (7)	0.3774 (2)	0.0490 (11)	
C5B	0.1362 (3)	0.5460 (6)	0.4248 (2)	0.0434 (10)	
C6	0.0274 (5)	0.6961 (8)	0.3344 (3)	0.0630 (14)	
H6	−0.0342	0.7004	0.3041	0.076*	
C7	0.0992 (5)	0.8348 (8)	0.3360 (3)	0.0705 (15)	
H7	0.0872	0.9309	0.3057	0.085*	
C8	0.1889 (5)	0.8350 (7)	0.3816 (3)	0.0622 (13)	
H8	0.2368	0.9312	0.3822	0.075*	
C8A	0.2087 (4)	0.6929 (6)	0.4269 (2)	0.0486 (11)	
C9	0.2985 (4)	0.6875 (6)	0.4764 (3)	0.0507 (11)	
H9	0.3459	0.7843	0.4795	0.061*	
C10	0.3174 (4)	0.5475 (6)	0.5188 (3)	0.0485 (10)	
H10	0.3766	0.5510	0.5511	0.058*	
C10A	0.2495 (3)	0.3931 (6)	0.5161 (2)	0.0408 (9)	
C11	0.3678 (4)	0.2211 (6)	0.6034 (3)	0.0477 (10)	
C12	0.5016 (4)	0.0193 (6)	0.6460 (3)	0.0521 (11)	
H12A	0.4762	0.0247	0.6957	0.063*	
H12B	0.5622	0.1027	0.6400	0.063*	
C13	0.5397 (4)	−0.1636 (6)	0.6279 (3)	0.0537 (11)	
H13A	0.4753	−0.2406	0.6255	0.064*	
H13B	0.5739	−0.1623	0.5804	0.064*	
C14	0.6204 (3)	−0.2380 (6)	0.6806 (2)	0.0450 (9)	
C15	0.7168 (4)	−0.1566 (7)	0.7033 (3)	0.0582 (12)	
H15	0.7400	−0.0471	0.6864	0.070*	
C16	0.6081 (4)	−0.4007 (7)	0.7125 (3)	0.0553 (11)	
H16	0.5479	−0.4733	0.7017	0.066*	
S1	0.7874 (4)	−0.2729 (6)	0.7637 (3)	0.0658 (13)	0.523 (7)
C17	0.6862 (17)	−0.450 (3)	0.7596 (15)	0.066 (4)	0.523 (7)
H17	0.6883	−0.5554	0.7854	0.079*	0.523 (7)
S1A	0.7088 (5)	−0.4534 (8)	0.7678 (4)	0.0682 (16)	0.477 (7)
C17A	0.782 (2)	−0.250 (3)	0.7479 (15)	0.071 (5)	0.477 (7)
H17A	0.8515	−0.2173	0.7653	0.085*	0.477 (7)

### Atomic displacement parameters (Å^2^)

**Table:**

	*U*^11^	*U*^22^	*U*^33^	*U*^12^	*U*^13^	*U*^23^
O1	0.070 (2)	0.0563 (19)	0.071 (2)	0.0105 (17)	−0.0173 (18)	−0.0139 (18)
O2	0.0506 (16)	0.0516 (16)	0.0507 (16)	0.0079 (14)	−0.0132 (14)	−0.0026 (15)
C1	0.040 (2)	0.047 (2)	0.039 (2)	0.0071 (18)	0.0023 (18)	−0.0015 (18)
C2	0.049 (3)	0.048 (3)	0.053 (3)	0.000 (2)	0.010 (2)	0.005 (2)
C3	0.042 (2)	0.049 (2)	0.056 (3)	−0.0065 (19)	0.001 (2)	−0.002 (2)
C3A	0.038 (2)	0.050 (2)	0.044 (2)	−0.0021 (17)	0.0097 (18)	−0.0046 (19)
C3B	0.0375 (19)	0.043 (2)	0.037 (2)	0.0057 (16)	0.0066 (17)	−0.0062 (17)
C4	0.037 (2)	0.066 (3)	0.054 (3)	−0.006 (2)	0.0048 (19)	−0.007 (2)
C5	0.044 (2)	0.077 (3)	0.044 (2)	0.006 (2)	−0.006 (2)	−0.007 (2)
C5A	0.049 (2)	0.061 (3)	0.037 (2)	0.014 (2)	0.0033 (19)	−0.005 (2)
C5B	0.046 (2)	0.050 (2)	0.0343 (18)	0.0089 (19)	0.0069 (18)	−0.0044 (19)
C6	0.073 (3)	0.070 (3)	0.045 (3)	0.019 (3)	−0.008 (2)	−0.001 (2)
C7	0.103 (4)	0.056 (3)	0.052 (3)	0.018 (3)	−0.005 (3)	0.005 (2)
C8	0.080 (3)	0.049 (3)	0.058 (3)	0.003 (2)	0.007 (3)	0.005 (2)
C8A	0.054 (3)	0.045 (2)	0.046 (2)	0.0068 (19)	0.010 (2)	−0.004 (2)
C9	0.049 (2)	0.043 (2)	0.060 (3)	−0.0017 (19)	0.004 (2)	−0.003 (2)
C10	0.043 (2)	0.048 (3)	0.054 (2)	0.0007 (19)	0.001 (2)	−0.006 (2)
C10A	0.0352 (19)	0.046 (2)	0.041 (2)	0.0034 (17)	0.0052 (17)	−0.0051 (18)
C11	0.046 (2)	0.050 (2)	0.047 (2)	0.0033 (19)	0.001 (2)	0.000 (2)
C12	0.053 (2)	0.053 (2)	0.051 (3)	0.004 (2)	−0.010 (2)	0.002 (2)
C13	0.056 (2)	0.056 (3)	0.049 (2)	0.005 (2)	−0.006 (2)	0.003 (2)
C14	0.0417 (17)	0.0518 (19)	0.041 (2)	0.0043 (14)	0.0039 (16)	0.0031 (16)
C15	0.0474 (19)	0.062 (2)	0.065 (3)	−0.0019 (17)	−0.0052 (19)	0.010 (2)
C16	0.056 (2)	0.055 (2)	0.055 (2)	−0.0008 (17)	−0.002 (2)	0.0091 (18)
S1	0.0549 (17)	0.071 (2)	0.071 (3)	0.0106 (14)	−0.0107 (16)	0.0112 (17)
C17	0.065 (5)	0.070 (5)	0.063 (7)	0.004 (4)	−0.009 (5)	0.011 (5)
S1A	0.069 (3)	0.067 (2)	0.068 (3)	0.0100 (18)	−0.016 (2)	0.0109 (19)
C17A	0.063 (5)	0.073 (5)	0.076 (10)	0.007 (4)	−0.014 (6)	0.012 (6)

### Geometric parameters (Å, º)

**Table:**

O1—C11	1.193 (6)	C8—C8A	1.386 (7)
O2—C11	1.330 (5)	C8—H8	0.9300
O2—C12	1.438 (5)	C8A—C9	1.417 (6)
C1—C2	1.388 (7)	C9—C10	1.339 (7)
C1—C10A	1.395 (6)	C9—H9	0.9300
C1—C11	1.484 (6)	C10—C10A	1.427 (7)
C2—C3	1.373 (6)	C10—H10	0.9300
C2—H2	0.9300	C12—C13	1.498 (7)
C3—C3A	1.375 (7)	C12—H12A	0.9700
C3—H3	0.9300	C12—H12B	0.9700
C3A—C3B	1.411 (6)	C13—C14	1.488 (6)
C3A—C4	1.437 (7)	C13—H13A	0.9700
C3B—C5B	1.412 (6)	C13—H13B	0.9700
C3B—C10A	1.432 (6)	C14—C16	1.375 (6)
C4—C5	1.332 (7)	C14—C15	1.378 (6)
C4—H4	0.9300	C15—C17A	1.341 (19)
C5—C5A	1.427 (7)	C15—S1	1.656 (6)
C5—H5	0.9300	C15—H15	0.9300
C5A—C6	1.391 (7)	C16—C17	1.334 (19)
C5A—C5B	1.409 (6)	C16—S1A	1.635 (6)
C5B—C8A	1.413 (7)	C16—H16	0.9300
C6—C7	1.360 (9)	S1—C17	1.814 (16)
C6—H6	0.9300	C17—H17	0.9300
C7—C8	1.369 (8)	S1A—C17A	1.810 (17)
C7—H7	0.9300	C17A—H17A	0.9300
			
C11—O2—C12	116.5 (3)	C8A—C9—H9	119.0
C2—C1—C10A	120.4 (4)	C9—C10—C10A	122.1 (4)
C2—C1—C11	117.7 (4)	C9—C10—H10	118.9
C10A—C1—C11	121.9 (4)	C10A—C10—H10	118.9
C3—C2—C1	121.3 (4)	C1—C10A—C10	124.5 (4)
C3—C2—H2	119.4	C1—C10A—C3B	118.2 (4)
C1—C2—H2	119.4	C10—C10A—C3B	117.2 (4)
C2—C3—C3A	120.5 (4)	O1—C11—O2	123.9 (4)
C2—C3—H3	119.7	O1—C11—C1	125.7 (4)
C3A—C3—H3	119.7	O2—C11—C1	110.4 (4)
C3—C3A—C3B	119.9 (4)	O2—C12—C13	106.9 (4)
C3—C3A—C4	121.5 (4)	O2—C12—H12A	110.3
C3B—C3A—C4	118.6 (4)	C13—C12—H12A	110.3
C3A—C3B—C5B	120.4 (4)	O2—C12—H12B	110.3
C3A—C3B—C10A	119.7 (4)	C13—C12—H12B	110.3
C5B—C3B—C10A	119.9 (4)	H12A—C12—H12B	108.6
C5—C4—C3A	120.6 (4)	C14—C13—C12	113.7 (4)
C5—C4—H4	119.7	C14—C13—H13A	108.8
C3A—C4—H4	119.7	C12—C13—H13A	108.8
C4—C5—C5A	122.2 (4)	C14—C13—H13B	108.8
C4—C5—H5	118.9	C12—C13—H13B	108.8
C5A—C5—H5	118.9	H13A—C13—H13B	107.7
C6—C5A—C5B	118.7 (5)	C16—C14—C15	111.2 (4)
C6—C5A—C5	122.7 (5)	C16—C14—C13	123.4 (4)
C5B—C5A—C5	118.6 (4)	C15—C14—C13	125.4 (4)
C5A—C5B—C3B	119.7 (4)	C17A—C15—C14	116.2 (10)
C5A—C5B—C8A	119.5 (4)	C14—C15—S1	113.5 (4)
C3B—C5B—C8A	120.9 (4)	C14—C15—H15	123.3
C7—C6—C5A	121.2 (5)	S1—C15—H15	123.3
C7—C6—H6	119.4	C17—C16—C14	117.1 (10)
C5A—C6—H6	119.4	C14—C16—S1A	114.1 (4)
C6—C7—C8	120.9 (5)	C17—C16—H16	121.4
C6—C7—H7	119.6	C14—C16—H16	121.4
C8—C7—H7	119.6	C15—S1—C17	91.3 (8)
C7—C8—C8A	120.5 (5)	C16—C17—S1	106.9 (14)
C7—C8—H8	119.7	C16—C17—H17	126.5
C8A—C8—H8	119.7	S1—C17—H17	126.5
C8—C8A—C5B	119.2 (4)	C16—S1A—C17A	91.4 (8)
C8—C8A—C9	123.0 (5)	C15—C17A—S1A	106.9 (14)
C5B—C8A—C9	117.8 (4)	C15—C17A—H17A	126.5
C10—C9—C8A	122.0 (4)	S1A—C17A—H17A	126.5
C10—C9—H9	119.0		
			
C10A—C1—C2—C3	−1.3 (6)	C2—C1—C10A—C10	−179.4 (4)
C11—C1—C2—C3	179.7 (4)	C11—C1—C10A—C10	−0.4 (6)
C1—C2—C3—C3A	2.7 (7)	C2—C1—C10A—C3B	−1.4 (6)
C2—C3—C3A—C3B	−1.4 (6)	C11—C1—C10A—C3B	177.6 (4)
C2—C3—C3A—C4	176.4 (4)	C9—C10—C10A—C1	174.9 (4)
C3—C3A—C3B—C5B	177.4 (4)	C9—C10—C10A—C3B	−3.1 (6)
C4—C3A—C3B—C5B	−0.5 (5)	C3A—C3B—C10A—C1	2.7 (5)
C3—C3A—C3B—C10A	−1.3 (6)	C5B—C3B—C10A—C1	−176.0 (4)
C4—C3A—C3B—C10A	−179.2 (4)	C3A—C3B—C10A—C10	−179.2 (4)
C3—C3A—C4—C5	−177.9 (4)	C5B—C3B—C10A—C10	2.1 (5)
C3B—C3A—C4—C5	−0.1 (6)	C12—O2—C11—O1	4.5 (7)
C3A—C4—C5—C5A	0.4 (7)	C12—O2—C11—C1	−174.8 (4)
C4—C5—C5A—C6	178.7 (5)	C2—C1—C11—O1	−140.3 (5)
C4—C5—C5A—C5B	−0.2 (6)	C10A—C1—C11—O1	40.7 (7)
C6—C5A—C5B—C3B	−179.4 (4)	C2—C1—C11—O2	38.9 (5)
C5—C5A—C5B—C3B	−0.4 (6)	C10A—C1—C11—O2	−140.0 (4)
C6—C5A—C5B—C8A	−0.6 (6)	C11—O2—C12—C13	−178.3 (4)
C5—C5A—C5B—C8A	178.3 (4)	O2—C12—C13—C14	−170.9 (4)
C3A—C3B—C5B—C5A	0.8 (5)	C12—C13—C14—C16	128.8 (5)
C10A—C3B—C5B—C5A	179.4 (4)	C12—C13—C14—C15	−52.1 (6)
C3A—C3B—C5B—C8A	−178.0 (4)	C16—C14—C15—C17A	3.1 (17)
C10A—C3B—C5B—C8A	0.7 (6)	C13—C14—C15—C17A	−176.1 (17)
C5B—C5A—C6—C7	2.1 (7)	C16—C14—C15—S1	−1.9 (6)
C5—C5A—C6—C7	−176.8 (5)	C13—C14—C15—S1	178.9 (5)
C5A—C6—C7—C8	−2.0 (8)	C15—C14—C16—C17	1.6 (16)
C6—C7—C8—C8A	0.4 (8)	C13—C14—C16—C17	−179.2 (16)
C7—C8—C8A—C5B	1.0 (7)	C15—C14—C16—S1A	0.1 (6)
C7—C8—C8A—C9	−178.5 (5)	C13—C14—C16—S1A	179.3 (5)
C5A—C5B—C8A—C8	−0.9 (6)	C14—C15—S1—C17	1.3 (11)
C3B—C5B—C8A—C8	177.9 (4)	C14—C16—C17—S1	−1 (2)
C5A—C5B—C8A—C9	178.7 (4)	C15—S1—C17—C16	−0.4 (18)
C3B—C5B—C8A—C9	−2.6 (6)	C14—C16—S1A—C17A	−2.2 (12)
C8—C8A—C9—C10	−178.8 (5)	C14—C15—C17A—S1A	−5 (2)
C5B—C8A—C9—C10	1.7 (6)	C16—S1A—C17A—C15	3.8 (19)
C8A—C9—C10—C10A	1.2 (7)		

### Hydrogen-bond geometry (Å, º)

**Table:**

*D*—H···*A*	*D*—H	H···*A*	*D*···*A*	*D*—H···*A*
C16—H16···O1^i^	0.93	2.55	3.448 (6)	161
C13—H13*B*···*Cg*3^ii^	0.97	2.86	3.776 (5)	155

Symmetry codes: (i) *x*, *y*−1, *z*; (ii) *x*+1/2, −*y*+1/2, *z*.
