# Supplementary figures and images for: Crystal structure of 2-(thio­phen-3-yl)ethyl pyrene-1-carboxyl­ate
Source: Acta Crystallogr E Crystallogr Commun. 2015 Nov 11;71(Pt 12):o926–7. doi: 10.1107/S2056989015020873 (PMC4719884; doi:10.1107/S2056989015020873)

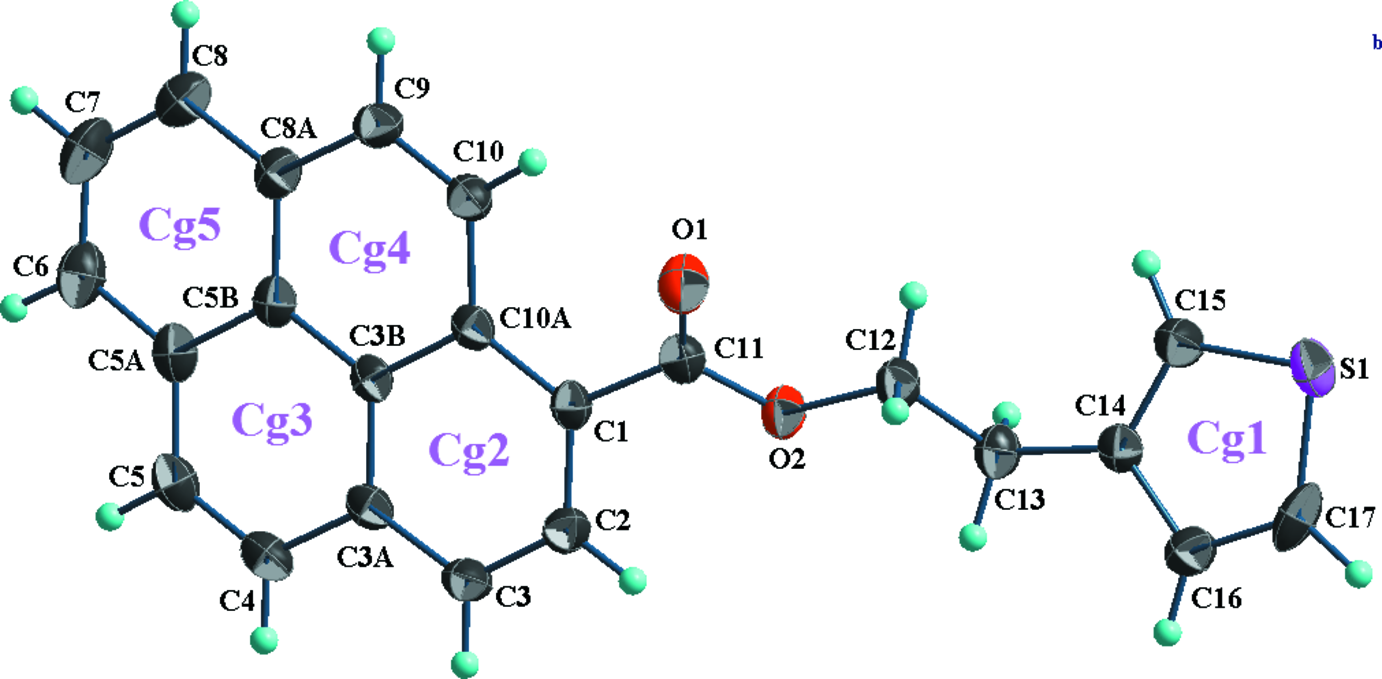

Supplement: Supplementary file 4 [file e-71-0o926-fig1.tif]

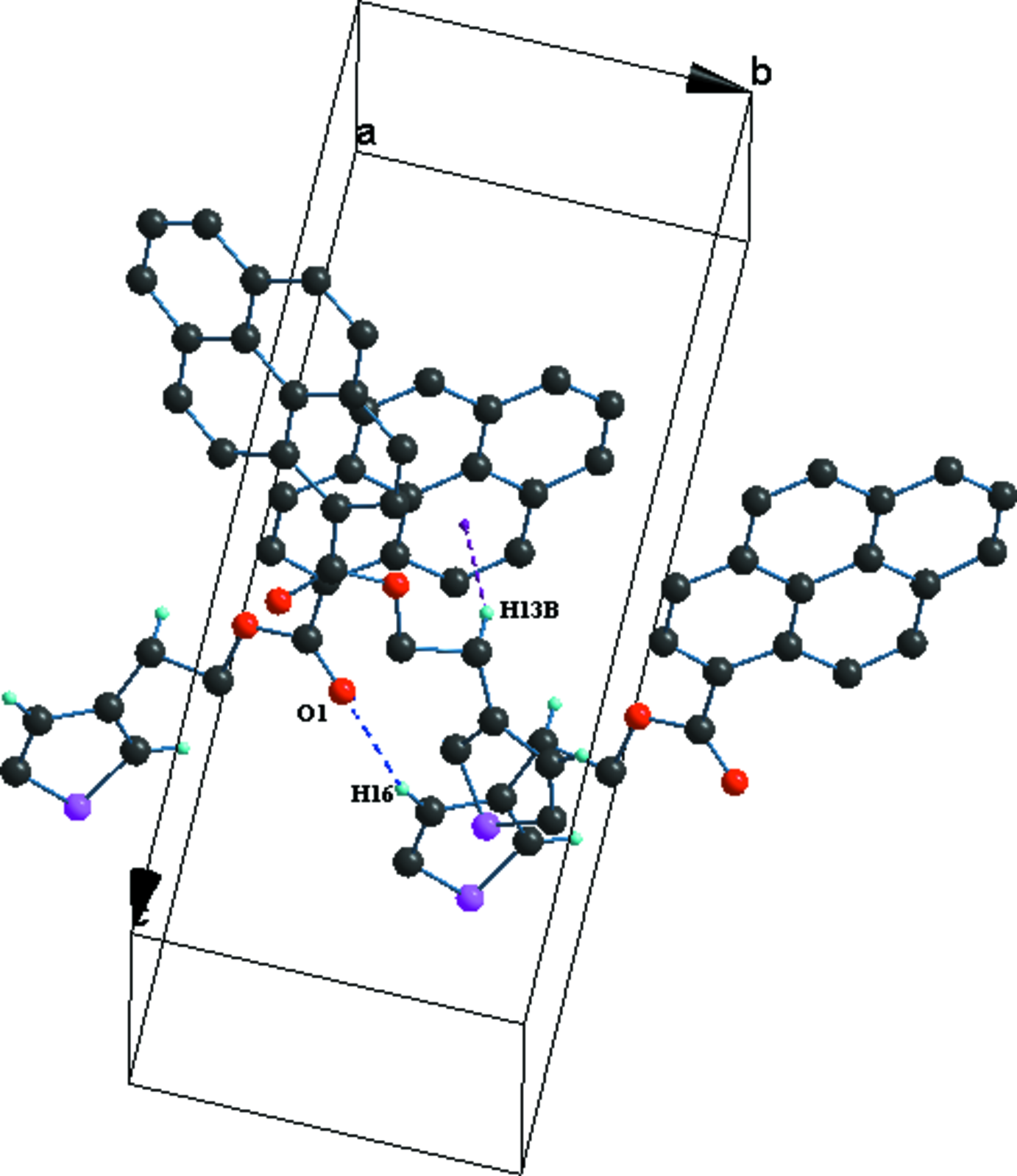

Supplement: Supplementary file 5 [file e-71-0o926-fig2.tif]

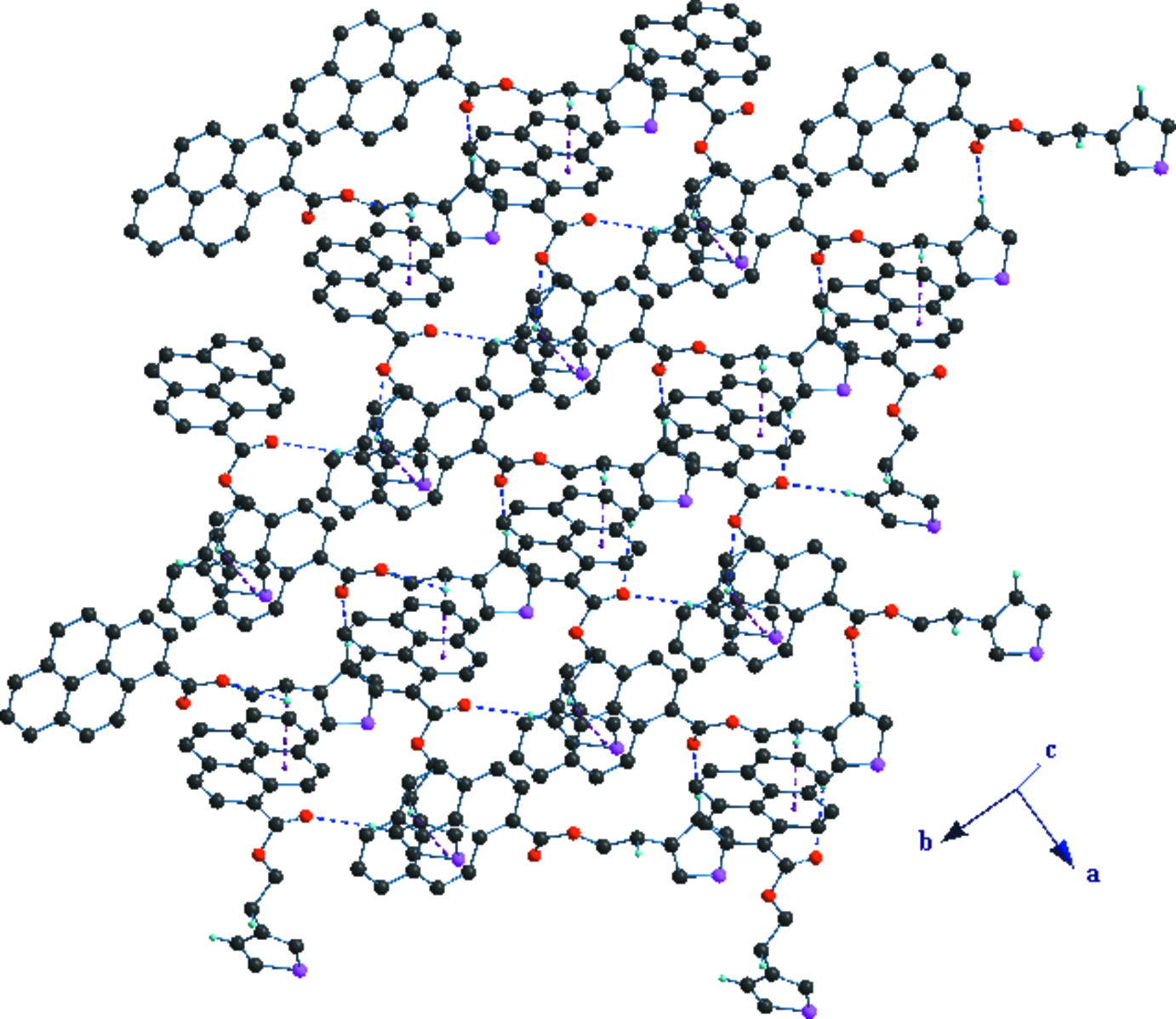

Supplement: Supplementary file 6 [file e-71-0o926-fig3.tif]

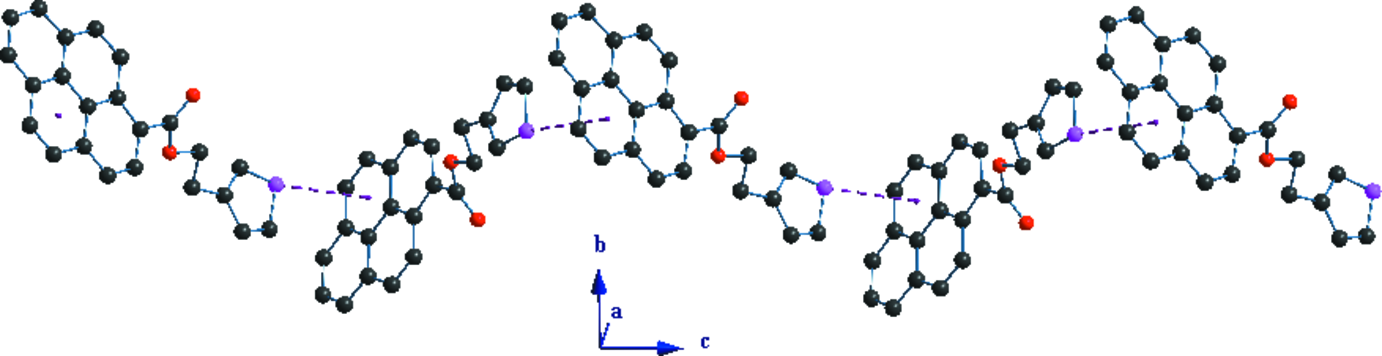

Supplement: Supplementary file 7 [file e-71-0o926-fig4.tif]
